# Visual Outcomes of Microkeratome-Assisted Anterior Lamellar Keratoplasty in Keratoconus: 5-Year Results

**DOI:** 10.1155/2022/3885524

**Published:** 2022-06-10

**Authors:** Ahmed Rashad Ashor, Mohamed-Sameh H. El-Agha, Mohamed Waleed Nagaty, Essam Abdel Ghaffar Darwish

**Affiliations:** Department of Ophthalmology, Faculty of Medicine, Cairo University, Cairo, Egypt

## Abstract

**Purpose:**

To report 5-year results of microkeratome-assisted anterior lamellar keratoplasty (MK-ALK) in cases of keratoconus.

**Methods:**

Patients with advanced keratoconus and the thinnest corneal location 300 *μ* or more were recruited. A Carriazo–Barraquer microkeratome was used to remove a 200-*μ* cap from the recipient cornea, and to prepare a 300-*μ* anterior stromal graft from a donor cornea. A full-thickness crescentic incision was made in the posterior stromal recipient bed using a 6.5-mm suction trephine. The donor was sutured to the recipient bed using interrupted nylon sutures. The minimum follow-up was five years.

**Results:**

Twelve eyes of 12 patients were included. The mean age was 26 ± 8 years. None of the patients required conversion to penetrating keratoplasty. Mean logMAR uncorrected and best spectacle-corrected visual acuity, respectively, improved from 1.56 ± 0.24 and 1.18 ± 0.32 preoperatively, to 0.63 ± 0.38 and 0.18 ± 0.12, five years after surgery (*P*=0.001for both). There was also a statistically significant reduction of mean manifest spherical equivalent, refractive cylinder, and mean keratometry readings. Posterior stromal striations occurred in all patients immediately after surgery but resolved after a maximum of 3 months. At five-years, anterior segment optical coherence tomography revealed a clear interface in all cases and a mean graft thickness of 328 ± 27 *μ*.

**Conclusion:**

MK-ALK is a safe and effective procedure for advanced keratoconus. Where feasible, it may be the best choice for patients at high risk of poor outcomes with penetrating keratoplasty, such as young patients with atopic keratoconjunctivitis or Down's syndrome.

## 1. Introduction

Penetrating keratoplasty (PKP) in advanced keratoconus eyes is generally very favorable, but graft rejection, induction of vision-limiting astigmatism, and late astigmatic progression are not infrequent [1–4]. Since in these cases the corneal endothelium is usually intact, with good cell count even after acute hydrops, there has been a recent trend to perform lamellar keratoplasty (LK) in keratoconic eyes [5, 6].

Anterior lamellar keratoplasty (ALK) targets partial or lamellar replacement of diseased corneal tissue while the posterior stroma is preserved [7]. The advantages of ALK include the absence of the risk of endothelial graft rejection, retention of structural integrity, and reduction of potential intraoperative complications associated with open sky procedures [8]. Improved instrumentation, surgical techniques, and automation have improved surgical efficiency and visual outcomes following ALK surgery [9]. Studies confirm that ALK visual outcomes are comparable to those of PK [10].

Deep anterior lamellar keratoplasty (DALK) has the advantage of reducing the risk of interface haze or scarring, but generally, it is technically difficult, and the risk of conversion to penetrating keratoplasty is usually high [11].

The advent of microkeratomes (both manual and automated) has allowed anterior lamellar keratoplasty to be readily performed in a reproducible manner [12]. Microkeratomes are adapted from those available for laser in situ keratomileusis (LASIK) and are commercially available, although it is desirable to have a microkeratome with variable depth plates [13]. In addition, artificial anterior chambers are available for use with corneoscleral buttons if whole eyes are not available [13]. The automated microkeratome (without the stop screw so that a free flap is produced) is used to cut the donor lenticule as well as the corneal disc in the recipient eye [14]. The thickness of the cut can be adjusted by choosing the proper plate size (up to 450 *μ*m) [14].

The aim of this study is to evaluate visual and refractive outcomes after microkeratome-assisted ALK for keratoconus. Secondary outcome measures include the incidence of interface haze or other complications, and the predictability and consistency of anterior lamellar graft thickness, as measured by anterior segment optical coherence tomography (AS-OCT).

## 2. Materials and Methods

Twelve eyes of 12 consecutive patients in this study were recruited from the Ophthalmology clinic at Kasr Al Ainy Hospital, Cairo University, during the period from October 2014 till April 2016. We included patients with advanced keratoconus who were intolerant to contact lenses (CL) and had best spectacle-corrected visual acuity (BSCVA) less than 1.0 LogMAR. We excluded eyes with the thinnest corneal point less than 300 *μ* and those with an interrupted Descemet's membrane (or history of acute hydrops). The study was approved by the Ethical Committee of Kasr Al Ainy School of Medicine, Cairo University. All patients included in this study signed an informed consent for the procedure conforming with the declarations of Helsinki.

Preoperatively and in different postoperative visits (1, 3, 6 months and then 1 year and 5 years) the following were recorded:

1. Uncorrected distance visual acuity (UCDVA)
2. Manifest refraction and best spectacle-corrected visual acuity (BSCVA)
3. Slit lamp examination of the anterior and posterior segments with full dilatation and tonometry

Scheimpflug imaging with the Sirius Scheimpflug Analyzer (CSO, Costruzione Strumenti Oftalmici, Florence, Italy) was performed preoperatively and 1 month, 3 months, 1 year and 5 years postoperatively to determine corneal thickness and simulated keratometric readings.

Anterior segment optical coherence tomography (AS-OCT) was performed using DRI OCT Triton, Swept Source OCT (Topcon Corporation, Tokyo, Japan) at one month, 6 months, one year, and five years after the intervention, to assess graft thickness, thickness of the residual posterior stromal bed, posterior stromal striations, and interface haze of the grafted cornea. Anterior lamellar keratoplasty (ALK) was performed using the Moria artificial anterior chamber and Carriazo–Barraquer microkeratome (Moria/Microtek, Inc, Doylestown, Pennsylvania) to harvest the graft and the same microkeratome to remove the required anterior lamellar cap from the recipient cornea.

### 2.1. Preparation of Donor Graft

**Figure 1:**
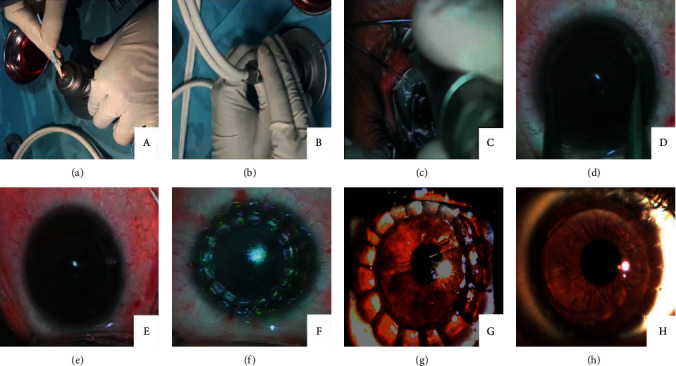
(a) Removal of epithelium from the graft using microsponge, (b) cutting the graft using 300 *μ* microkeratome head, (c) preparation of recipient cornea using 200 *μ* microkeratome head, (d) measuring the diameter of residual recipient bed using a caliper, (e) recipient stromal bed after incomplete full thickness trephination using 6.5 mm suction trephine, (f) suturing of the donor graft using 16 interrupted nylon 10-0, (g) posterior stromal striations in case no. 5 at early postoperative period, and (h) complete disappearance of posterior stromal striation and a clear cornea of the same patient in (g), one year after surgery.

Graft preparation was performed at the Cairo University Eye Bank. The corneal graft was placed over the artificial anterior chamber, ensuring adequate fitting and graft centration. The pressure of the anterior chamber was maintained by a balanced salt solution (BSS) infusing from a bottle elevated to a height of 215 cm to ensure maximum pressure elevation. Because the graft would ultimately be covered with recipient epithelium, the donor epithelium was removed with a dry microsponge to ensure the inclusion of more stroma in the donor graft by the microkeratome cut. After that, the 300 *μ*m head was used to fashion the donor's cornea with a maximum diameter of 10 mm using the microkeratome without a stop (Figures 1(a) and 1(b)).

### 2.2. Preparation of Recipient Cornea

The microkeratome suction ring (size +2.0) was applied over the recipient cornea to create a vacuum, then the microkeratome (200 *μ* head) was placed onto the suction ring, and activated to make a lamellar cut in the recipient cornea. Thus, a free corneal cap 200 *μ* in thickness was removed from the recipient (Figure 1(c)).

After measuring the diameter of the residual recipient bed using a caliper (Figure 1(d)), the oversized fashioned donor's graft was trephined with a Katena corneal punch (Katena Products, Inc, Danville, New Jersey) to be the same diameter as that of the recipient bed.

Then, a Barron suction trephine (Katena Products, Inc, Danville, New Jersey) with a diameter of 6.5 mm was centered on the pupil over the recipient residual stromal bed and the blade was advanced until aqueous was seen to start escaping, indicating penetration of the stromal bed. Suction was then immediately released (Figure 1(e)). This incomplete full-thickness trephination in the bed is important to neutralize the effect of residual recipient stroma that preserves a “keratoconus memory,” causing excessive steepening and irregularity of the final corneal contour [14]. Then the lamellar graft was sutured in place using 16 interrupted 10-0 nylon sutures, and knots were buried (Figure 1(f)). Finally, an eye patch was applied over the tobramycin and dexamethasone ointment (Tobradex®; Alcon, Inc). Postoperatively the patients received tobramycin 0.3% and dexamethasone 0.1% ophthalmic suspension (Tobradex®; Alcon, Inc) five times daily tapered over 6 weeks. In all patients, all sutures were removed 6 months following the procedure.

All statistical calculations were performed using the computer program SPSS (Statistical Package for the Social Science; SPSS Inc., Chicago, IL, USA) version 23, by which numerical data in preoperative and different postoperative follow-up visits were tested and compared using the Friedman test and the Wilcoxon signed-rank test “Post hoc”. *P*values less than 0.05 were considered statistically significant.

## 3. Results

This study included 12 eyes of 12 patients, 6 males and 6 females. The mean age of the patients was 26.1 ± 8.2 years (range: 15 to 42 years). All patients completed at least five years of follow-up.

Table 1 shows mean UCDVA, BSCVA, manifest spherical equivalent (MSE), average simulated keratometry (Kmean), and thinnest corneal location thickness in preoperative and postoperative follow-up visits. Friedman test was used to test the hypothesis that there were no differences between the means of repeated measures within each variable. The *P*value for each test is less than 0.05, which indicates the existence of significant differences in each variable in the different time measures. Based on the Friedman test results a post hoc test called “Wilcoxon signed-rank test” had to be performed to check the pair wise differences. The results of pairwise comparisons were summarized using lowercase letters in a way that the time points that do not share a letter have significantly different mean values.

From Table 1, we can see that there was a statistically significant improvement in both UCDVA and BSCVA. There was also a statistically significant reduction in mean MSE and mean Kmean and an increase in the thinnest corneal location thickness. All of this represents a normalization of corneal anatomy.

An improvement in manifest cylinder was also noticed, the mean manifest cylinder was −6.65 ± 3.23 D preoperative and -2.52 ± 2.03 D at five years postoperatively (*P*=0.0001).

The mean central thickness of the anterior lamellar graft (epithelium + stroma), as measured by AS-OCT, was 328 *μ* one month after surgery, and was stable at this value for the rest of the follow-up period (Table 1). The mean residual posterior stromal thickness (under the anterior lamellar graft) was also found to stabilize around 6 months at an average value of approximately 158 *μ* (Table 1).

Since both the donor cap and the removed recipient cap were created by the same microkeratome, both were meniscus shaped (thinner in the center), with sloping edges (despite we cut a small peripheral portion of the donor cap after being created by the microkeratome to be as the same diameter as the measured recipient bed). This resulted in excellent apposition at the peripheral graft-host junction as well as a smooth graft-host interface, which was seen in all cases.

### 3.1. Difficulties and Complications of the Procedure

#### 3.1.1. During Preparation of the Donor Graft

In a single case (8.33%), a mismatch between the donor cap size (8.5 mm) and the recipient bed (9.0 mm) was noticed. This resulted in a flat cornea as well as a shallow anterior chamber in the early postoperative period. By the end of the first year after surgery, the corneal curvature normalized as did the anterior chamber depth.

#### 3.1.2. Intraoperative Difficulties

In one eye (8.33%), a narrow palpebral fissure made it very difficult to fully insert the suction ring within the surgical speculum. This was managed by the removal of the speculum to create more space for the insertion of the suction ring, and a strabismus hook was additionally needed to retract the lower eye lid. The surgery subsequently proceeded without further difficulties.

#### 3.1.3. Postoperative Complications

*(1) Posterior Stromal Striations*. All eyes (100%) had significant posterior stromal striations in the early postoperative period due to a redundant posterior lamella induced by the crescentic relaxing incision created with the suction trephine. In all cases, these striae completely disappeared after 12 ± 3.24 weeks (Figures 1(g) and 1(h)).

*(2) Ring of Scarring at the Site of Trephination in the Posterior Stromal Bed*. This occurred in 6 eyes (50%), but was away from the visual axis and did not affect vision.

*(3) Rejection*. Two eyes (16.66%) developed stromal rejection, manifesting as subepithelial and stromal haze associated with ciliary injection and photophobia, as well as diminution of vision, starting around 5–6 months after surgery. Total recovery of the cornea was achieved by topical prednisolone 1% topical eye drops every two hours followed by gradual tapering of the drops over 6 weeks.

*(4) Epithelial Defect*. In one eye (8.33%), a small epithelial defect (1 mm in diameter) developed 8 weeks after the surgery due to a flat cornea which prevented proper rewetting of the central cornea. Complete healing of the defect was achieved after one week of frequent preservative-free lubricant eye drops and eye patching.

*(5) Severe Postpperative Myopia*. This occurred in one eye (8.33%); the patient was 45 years old and had preexisting axial myopia (−14D myopia and axial length was 29.5 mm). This was managed by refractive clear lens exchange.

*(6) Fungal Keratitis in the Graft*. A single eye (8.33%) developed keratitis 4 weeks after surgery in the nasal aspect of the graft associated with a localized epithelial defect and three loose nasal sutures. Scrapings were performed, but smears and cultures were negative. Three loose sutures in the area of the infiltration were removed. Based on the clinical findings of mild conjunctival injection, the absence of pain, the presence of a dry well-defined infiltrate, and a well-documented incidence of fungal keratitis in corneal transplant tissue prepared by a microkeratome [15], we made a clinical diagnosis of fungal keratitis. Topical antifungal fortified eye drops (Fluconazole 2 mg/ml, Diflucan®) were started immediately every 2 hours with the cessation of topical steroids. Complete resolution of the infection was achieved after 21 days, and the BSCVA reached 0.22 LogMAR at five years follow-up visit with a mild superficial opacity that was well-away from the visual axis. Refraction was (0.00/−1.50 x 80).

## 4. Discussion

Many authors have investigated the use of anterior lamellar stromal grafts, as both inlays and onlays, to treat keratoconus, thus avoiding the unnecessary replacement of healthy endothelium and eliminating the occurrence of endothelial rejection [5–14, 16–18]. The most popular of these techniques is big-bubble deep anterior lamellar keratoplasty (DALK) [19–22]. Although it achieves the best visual results, up to 30–40% of cases need conversion to PKP [23]. Manual layer-by-layer dissection of the recipient cornea is needed if a big bubble is not obtained, but this is relatively time-consuming, and conversion to PKP is always an imminent threat [23]. Femtosecond laser-assisted ALK has also been used in keratoconus but with poor results [24].

The technique we employed in this study aims to eliminate this heavy dependence on individual surgeon skill by providing a reproducible technique that is within the range of any surgeon who can perform microkeratome-assisted laser in situ keratomileusis (LASIK) and PKP. The technique was first described by Massimo Busin and colleagues in 2005, followed by a modified procedure that was published by the same group in 2012 [14, 25]. In the original technique, a 300-micron donor cap was placed as an onlay on the recipient's cornea after removing a 200-micron cap from the recipient [25]. With this technique, many cases developed excessive corneal steepening and 9 of 50 cases (18%) developed postoperative irregular astigmatism, presumably because of the persistence of the conical shape in the posterior stromal recipient bed [25]. To avoid this problem and aiming to restore normal corneal anatomy, Busin et al. published a modified technique, adding a central 6.5-mm, incomplete full-thickness trephination of the residual bed, created by a 6.5-mm suction trephine [14]. In our study, we performed this modification to collapse the cone in all our operated eyes.

Visual results after PKP do not differ substantially from those of our series [16, 26]; nevertheless, visual rehabilitation is typically longer after PKP, as stable refraction is achieved only after suture removal is completed, usually later than 12 months, as opposed to 6 months in our LK patients.

Our results are comparable with the outcomes reported in an interventional case series of keratoconus eyes that underwent 8 mm DALK using big bubble and manual dissection techniques (mean logMAR BSCVA 0.10 ± 1.18, *n* = 20, compared to 0.18 ± 0.12 in our series) [27]. The percentage of eyes achieving logMAR BSCVA >0.3 in our series was 91.6%, compared to 78–89% in other studies using various DALK techniques including pneumatic, manual, and viscoelastic assisted lamellar dissection [27–29]. Our five-year results are also consistent with the 10-year results of a large series employing the same technique by the Busin group. In their study, over 90% reached 0.3 logMAR BSCVA at 10 years [30].

Other aspects of visual acuity improvement in our study included a reduction of manifest spherical equivalent and manifest cylinder.

In terms of restoration of normal corneal anatomy, the procedure resulted in normalization of keratometry readings. An initial exaggerated flattening was seen one month after surgery (Table 1), which was corrected after suture removal. The procedure also resulted in augmentation of corneal thickness: in this study, the thinnest corneal thickness increased from a preoperative value of 365 ± 57 *μ* to 416.83 ± 40.14 *μ* five years after surgery (*P*=0.001).

**Figure 2:**
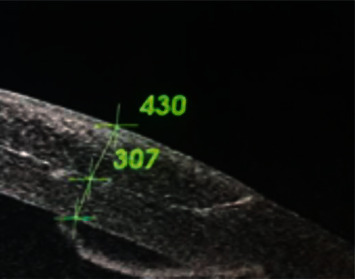
Showing excellent apposition at peripheral graft-host junction.

In both studies by Busin et al., there was no assessment by AS-OCT [14, 25]. Our AS-OCT studies revealed a reproducible graft thickness that was stable throughout five years of follow-up. Additionally, despite initial posterior stromal striations, OCT images show a perfectly smooth interface as early as 6 months after surgery, but typically by one year. This is probably an advantage of this procedure over femtosecond-assisted ALK, which typically produces irregular cuts in the posterior stroma of the donor and recipient corneas, which leads to interface haze [24]. In the microkeratome-enabled procedure, both the cuts in the donor and recipient are very smooth. OCT also revealed that there is very good apposition of the donor and recipient at the edge of the graft (Figure 2).

It can be argued that trephination of the recipient posterior stromal bed renders this surgery intraocular because of the full-thickness incision. However, the procedure does not involve any intraocular maneuvers, and we did not need any intracameral air to prevent a double anterior chamber.

In a series that included one hundred eyes, Busin et al. reported a group of postoperative complications such as double-chamber formation (3%), persistent epithelial defects (6%), wound dehiscence after suture removal (2%), and corticosteroid-induced posterior subcapsular cataract (2%) [14]. In our series, only one of the patients had delayed epithelial healing, but other complications reported by the Busin group were not encountered. However, 2 patients in our series developed stromal rejection that was reversed by topical steroids, and one patient developed fungal keratitis that was also successfully managed; all 3 patients ended up with good visual acuity.

We believe that microkeratome-assisted ALK with partial trephination of the posterior stromal recipient bed (cone collapse) has many advantages. Like other forms of ALK, it preserves host endothelium, which leads to a much higher probability of long-term graft survival. Unlike big-bubble DALK or manual layer-by-layer dissection, it is well within the skill range of the “average” surgeon. It is more reproducible than other forms of ALK (almost zero conversion to PKP). It provides a smoother interface than manual dissection or the femtosecond laser. The remainder of the donor can be used for Descemet-stripping automated endothelial keratoplasty (DSAEK), ultrathin DSAEK, predescemetic endothelial keratoplasty (PDEK), and Descemet's membrane endothelial keratoplasty (DMEK); with DALK, only DMEK is possible.

However, there are disadvantages to the procedure. It still requires sophisticated instrumentation which may not be readily available in all practice settings. Although cutting the recipient is relatively familiar to a surgeon who is skilled in microkeratome-assisted LASIK, cutting the donor using an artificial anterior chamber requires a significant learning curve (which may be obviated by delegating this portion of the procedure to an eye bank). The thickness of the thinnest corneal location has to be at least 300 *μ*, which is often not the case in many advanced cases of keratoconus requiring keratoplasty. Visually significant posterior stromal striations are inevitable but fortunately disappear within 3–6 months, and they do not affect the final visual outcome.

The main limitation of this study is the small sample size.

## 5. Conclusions

The findings of this study suggest that microkeratome-assisted ALK with cone collapse in cases of keratoconus has all the advantages of lamellar keratoplasty and provides favorable visual and anatomical outcomes with a low rate of complications. Wherever available, it may be the procedure of choice for keratoconus patients who are more prone to poor outcomes with penetrating keratoplasty, such as patients with atopic keratoconjunctivitis or young patients with Down's syndrome.

## Figures and Tables

**Table 1:** Preoperative and postoperative UCDVA, BSCVA, MSE, mean of simulated Kmean, thinnest location thickness, and postoperative mean central graft thickness and mean residual posterior stromal thickness.

	Preoperative	1 month	3 months	6 months	1 year	5 years	*P*value^*∗∗*^
UCDVA (logMAR)	1.56 ± 0.24^a^	0.92 ± 0.27^ab^	0.72 ± 0.29^b^	0.86 ± 0.30	0.74 ± 0.22^b^	0.62 ± 0.38^b^	<0.001
BSCVA (logMAR)	1.18 ± 0.32^a^	0.58 ± 0.24^b^	0.43 ± 0.18^b^	0.38 ± 0.12	0.27 ± 0.10^b^	0.18 ± 0.12^b^	<0.001
Manifest spherical equivalent (diopters)	−5.32 ± 2.39^a^	0.34 ± 2.11^b^	0.25 ± 2.69^bc^	−1.35 ± 3.0	−2.57 ± 2.85^ac^	−1.54 ± 2.13^abc^	<0.001
Mean of simulated Kmean (diopters)	57.6 ± 7.85^a^	42.76 ± 5.04^b^	46.00 ± 3.83^b^		46.99 ± 3.75^b^	46.56 ± 3.46^b^	<0.001
Thinnest location thickness (*μ*)	364.83 ± 57.4^a^	422.50 ± 45.11^b^	424.00 ± 40.87^b^		426.00 ± 37.64^b^	416.83 ± 40.14^b^	<0.001
Mean central graft thickness (*μ*m)		328.33 ± 20.834		328.9 ± 28.219	328.22 ± 26.682	328.22 ± 26.682	
Mean residual posterior stromal thickness (*μ*m)		151.67 ± 54.822		158 ± 53.281	158.33 ± 49.954	158.33 ± 49.954	

^*∗*^ Results (means ± standard deviation, Friedman test and Wilcoxon signed-rank test “Post hoc”) were organized in one table. ^*∗∗*^Friedman test, means that do not share a letter are significantly different.

## Data Availability

Derived data supporting the findings of this study are available from the corresponding author on request.

## References

[1] Mohammadpour M. (2007). Penetrating keratoplasty for keratoconus. *Ophthalmology*.

[2] Sray W. A., Cohen E. J., Rapuano C. J., Laibson P. R. (2002). Factors associated with the need for penetrating keratoplasty in keratoconus. *Cornea*.

[3] Gordon M. O., Steger-May K., Szczotka-Flynn L. (2006). Baseline factors predictive of incident penetrating keratoplasty in keratoconus. *American Journal of Ophthalmology*.

[4] Zadok D., Schwarts S., Marcovich A. (2005). Penetrating keratoplasty for keratoconus. *Cornea*.

[5] Patil M., Mehta J. S. (2020). Lamellar keratoplasty for advanced keratoconus. *Asia-Pacific Journal of Ophthalmology*.

[6] Fuest M., Mehta J. S. (2018). Strategies for deep anterior lamellar keratoplasty after hydrops in keratoconus. *Eye and Contact Lens: Science and Clinical Practice*.

[7] Espandar L., Carlson A. N. (2013). Lamellar keratoplasty: a literature review. *Journal of Ophthalmology*.

[8] Yeung S. N., Lichtinger A., Kim P., Amiran M. D., Rootman D. S. (2012). Retrospective contralateral study comparing deep anterior lamellar keratoplasty with penetrating keratoplasty: a patient’s perspective. *Canadian Journal of Ophthalmology*.

[9] Tan D. T., Mehta J. S. (2007). Future directions in lamellar corneal transplantation. *Cornea*.

[10] Henein C., Nanavaty M. A. (2017). Systematic review comparing penetrating keratoplasty and deep anterior lamellar keratoplasty for management of keratoconus. *Contact Lens and Anterior Eye*.

[11] Terry M. A. (2000). The evolution of lamellar grafting techniques over twenty-five years. *Cornea*.

[12] Alio J. L., Shah S., Barraquer C., Bilgihan K., Anwar M., Melles G. R. J. (2002). New techniques in lamellar keratoplasty. *Current Opinion in Ophthalmology*.

[13] Hafezi F., Mrochen M., Fankhauser F., Seiler T. (2003). Anterior lamellar keratoplasty with a microkeratome: a method for managing complications after refractive surgery. *Journal of Refractive Surgery*.

[14] Busin M., Scorcia V., Zambianchi L., Ponzin D. (2012). Outcomes from a modified microkeratome-assisted lamellar keratoplasty for keratoconus. *Archives of Ophthalmology*.

[15] Brothers K. M., Shanks R. M. Q., Hurlbert S., Kowalski R. P., Tu E. Y. (2017). Association between fungal contamination and eye bank-prepared endothelial keratoplasty tissue. *JAMA Ophthalmology*.

[16] Song Y., Zhang J., Pan Z. (2020). Systematic review and meta-analysis of clinical outcomes of penetrating keratoplasty versus deep anterior lamellar keratoplasty for keratoconus. *Experimental and Clinical Transplantation*.

[17] Lee W. B., Jacobs D. S., Musch D. C., Kaufman S. C., Reinhart W. J., Shtein R. M. (2009). Descemet’s stripping endothelial keratoplasty: safety and outcomes. *Ophthalmology*.

[18] Gadhvi K. A., Romano V., Fernández-Vega Cueto L., Aiello F., Day A. C., Allan B. D. (2019). Deep anterior lamellar keratoplasty for keratoconus: multisurgeon results. *American Journal of Ophthalmology*.

[19] Spadea L., Rosa V. D. (2016). Current techniques of lamellar keratoplasty for keratoconus. *Saudi Medical Journal*.

[20] Anwar M., Teichmann K. D. (2002). Big-bubble technique to bare descemet’s membrane in anterior lamellar keratoplasty. *Journal of Cataract and Refractive Surgery*.

[21] Fogla R., Padmanabhan P. (2006). Results of deep lamellar keratoplasty using the big-bubble technique in patients with keratoconus. *American Journal of Ophthalmology*.

[22] Fontana L., Parente G., Tassinari G. (2007). Clinical outcomes after deep anterior lamellar keratoplasty using the big-bubble technique in patients with keratoconus. *American Journal of Ophthalmology*.

[23] Koçluk Y., Alyamaç Sukgen E., Burcu A. (2017). Comparison of outcomes in patients who underwent deep anterior lamellar keratoplasty and those converted to penetrating keratoplasty. *Turkish Journal of Orthodontics*.

[24] Lu Y., Grisolia A. B., Ge Y. R. (2017). Comparison of femtosecond laser-assisted descemetic and predescemetic lamellar keratoplasty for keratoconus. *Indian Journal of Ophthalmology*.

[25] Busin M., Zambianchi L., Arffa R. (2005). Microkeratome-assisted lamellar keratoplasty for the surgical treatment of keratoconus. *Ophthalmology*.

[26] Choi J. A., Lee M. A., Kim M. S. (2014). Long-term outcomes of penetrating keratoplasty in keratoconus: analysis of the factors associated with final visual acuities. *International Journal of Ophthalmology*.

[27] Romano V., Iovieno A., Parente G., Soldani A. M., Fontana L. (2015). Long-term clinical outcomes of deep anterior lamellar keratoplasty in patients with keratoconus. *American Journal of Ophthalmology*.

[28] Kubaloglu A., Sari E. S., Unal M. (2011). Long-term results of deep anterior lamellar keratoplasty for the treatment of keratoconus. *American Journal of Ophthalmology*.

[29] Feizi S., Javadi M. A., Jamali H., Mirbabaee F. (2010). Deep anterior lamellar keratoplasty in patients with keratoconus: big-bubble technique. *Cornea*.

[30] Yu A. C., Franco E., Caruso L. (2021). Ten-year outcomes of microkeratome-assisted lamellar keratoplasty for keratoconus. *British Journal of Ophthalmology*.

